# Data-Driven Eco-Efficiency Analysis and Improvement in the Logistics Industry in Anhui

**DOI:** 10.3390/ijerph20064810

**Published:** 2023-03-09

**Authors:** Shiqiang Sun, Yujia Liu

**Affiliations:** 1 School of Economics, Liaoning University, Shenyang 110036, China; 2 School of Business, Suzhou University, Suzhou 234000, China

**Keywords:** eco-efficiency, data-driven, sustainability, logistics

## Abstract

The ecological efficiency (eco-efficiency) of a regional logistics industry (RLI) is widely regarded as a key factor affecting sustainability of economic development, environmental protection, and resources utilization. This study applied a data-driven method to evaluate and increase the eco-efficiency of an RLI. Based on RLI-related data, which were converted into proper dimensionless indices, data envelopment analysis (DEA), which assumes that the decision-making units (DMUs) are in the situation of variable returns to scale, the Banker, Charnes, and Cooper (BCC) model, and Malmquist index model were used to assess the eco-efficiency of the RLI from both static and dynamic viewpoints. Then, a Tobit regression model was built to explore the factors that influence eco-efficiency. The effectiveness of this approach was verified by its application to an example from Anhui Province. This study has theoretical and practical value for the assessment and promotion of the ecological eco-efficiency of the RLI. We believe that our approach offers a powerful tool to assist logistics enterprises and local governments in coordinating the relationship between the RLI economy and the ecological environment, facilitating the drive to carbon neutrality.

## 1. Introduction

The logistics industry, the basis of national economies, is an essential link that connects production and sales [1]. With fierce competition in the global market, the rapid development of the RLI consumes a variety of energy sources and causes environmental pollution [2] while achieving economic sustainability. The “Sustainable Development Goals Report 2022”, published by the United Nations Environment Programme (UNEP) in 2022, indicated that chain reactions and interconnection of crises, such as the COVID-19 pandemic and climate change, place continued existence of human beings in danger [3]. To mitigate these risks and realize the aims of the Sustainable Development Goals (SDGs), maximizing the eco-efficiency potential of energy-intensive industries is needed. The research report “Roadmap to Corporate Carbon Neutrality”, co-authored by the Boston Consulting Group (BCG) and United Nations Compact (UNGC), focuses on energy-intensive industries and points out that the RLI should first conduct carbon inspections before conducting emissions reductions in order to lay the foundation for the sustainability of the industry [4]. Measuring the eco-efficiency of the RLI is the basis for achieving energy conservation and emission reduction, and research on the eco-efficiency of the RLI can provide theoretical support for the sustainable development (SD) of the economy.

Schaltegger and Sturn proposed the concept of eco-efficiency and calculated it as the ratio of maximum economic output to resource input [5]. The level of implementation of the essence of eco-efficiency is a valid measure of high-quality development. The academic community generally regards eco-efficiency as a type of input–output ratio when researching connotations [6,7,8,9]. The World Business Council for Sustainable Development (WBCSD) defines eco-efficiency as a product that meets the requirements of basic needs and also quality of life with a reasonable environmental impact and resource utilization [10]. The European Environment Agency defines eco-efficiency as obtaining more with fewer resources [11].

When establishing an indicator system, scholars worldwide mostly analyze input and output from the perspective of input and output products. The specific input indicators include those relating to humans, capital, research and development (R&D), petroleum, coal, and other non-renewable energy sources; output indicators can be divided into desirable and undesirable indicators, such as wastewater [12], waste gas, waste residue, energy consumption, and CO_2_ emissions, which cause the greenhouse effect [13,14,15,16,17]. Eco-efficiency index systems are widely used in the industrial [18], agricultural [19], and service industries [20]. Liu et al. [21] constructed an industrial production system efficiency evaluation model based on emergy to quantitatively assess industrial production efficiency. Rybaczewska [22] combined the joint application of life cycle assessment (LCA) and data envelopment analysis (DEA) to evaluate the agriculture eco-efficiency in Europe.

After establishing systematic measurement indicators, scholars began to use various methods to evaluate eco-efficiency, such as the DEA [23], undesired output slacks-based measure (SBM) model [24], and two-stage network DEA [25,26]. DEA is appropriate for multiple input and output efficiency evaluation; however, the pure DEA model ignores the expression relationship of some data. Wang et al. [27] argued that existing DEA models pay more attention to comparison of eco-efficiency values but simultaneously neglect radial and non-radial classifications of indicators. Therefore, she proposed a hybrid super-efficient DEA model to explore the low-efficiency problem of the eco-efficient industrial sector more comprehensively. Considering the environmental impacts and agricultural production more flexibly, Angulo-Meza et al. [28] studied complex agricultural production systems and selected the LCA + DEA method to assess eco-efficiency. Considering dynamic efficiency and static efficiency, the present study applied the DEA–Malmquist method to render eco-efficiency at different times comparable.

From the perspective of the influencing factors and improvement methods for the RLI, eco-efficiency must be jointly maintained by governments, enterprises, and consumers [29,30,31]. Scholars have previously made suggestions based on different perspectives after conducting empirical analyses [32,33]. Considering RLI practitioners, Laguir et al. [34] found that economic performance of enterprises is proportional to environmental performance by studying the performances of 232 French third-party logistics (3PL) providers. Therefore, logistics enterprises should prioritize eco-efficiency orientation and view it from a strategic perspective. Specifically, they can negotiate cooperatively with suppliers regarding recycling, reuse, and resource reduction. The government should increase publicity and promotion efforts; establish a cooperative R&D mechanism among logistics enterprises, scientific research units, and governments; and promote R&D and application of products with high levels of eco-efficiency. Chu [35] applied hierarchical moderated regression analysis to 165 Chinese 3PL providers to show that customer pressure is a vital driving force for logistics enterprise innovation and that customers’ environmental needs can prompt companies to formulate green innovation initiatives. Managers can empower technicians with greater autonomy to promote environmentally friendly products.

Based on a review of the existing research, RLI has significant potential for economic development but also large resource consumption, and coordination between them is a key issue. As such, three main following problems exist in improving eco-efficiency. (1) Evaluating the eco-efficiency of the RLI requires comprehensive consideration of social, economic, and environmental inputs, as well as desired and undesired outputs, and involves many variables at different time periods and with complex conversion relationships. How to calculate the eco-efficiency of the RLI accurately on a uniform scale remains to be studied in depth. (2) Most existing studies have collected data about the RLI at a national level and from the perspectives of the government and RLI practitioners. However, research results at this level do not fully reflect the industry scale of different regions, particularly from the aspect of eco-efficiency in less economically developed regions. Based on system modelling and data-driven research, research on improving the eco-efficiency of the RLI from different levels and perspectives to promote industrial SD is yet to be explored. (3) The eco-efficiency of a regional RLI is affected by various factors; therefore, quantifying the weights of each influencing factor based on regional heterogeneity is required.

Inspired by the ideas of Mirmozaffari et al. [36], Liu et al. [37], and Ma et al. [38], this research first developed a data-driven method for measurement and improvement in RLI eco-efficiency. Then, input and output data were collected from the aspects of economy, resources, and environment to evaluate the eco-efficiency of the RLI, and a targeted evaluation index system was constructed to provide unified measurement of the eco-efficiency of the RLI. After that, a “measurable and optimizable” eco-efficiency evaluation method for RLI was established through a data-driven approach, providing methodological support for objective evaluation. Moreover, the Tobit model was applied on identifying key factors and exploring the relationship between those factors and eco-efficiency, providing a significant basis for designing government policy. Finally, this research used a case study to verify the effectiveness of the approach. From a practical standpoint, the positive results of this research will provide a reference for logistics enterprises and production managers to quantify the inputs and outputs of enterprises objectively, coordinate economic performance and environmental protection, and improve the resource use efficiency and SD of the RLI.

## 2. Materials and Methods

### 2.1. Method Flow

**Figure 1:**
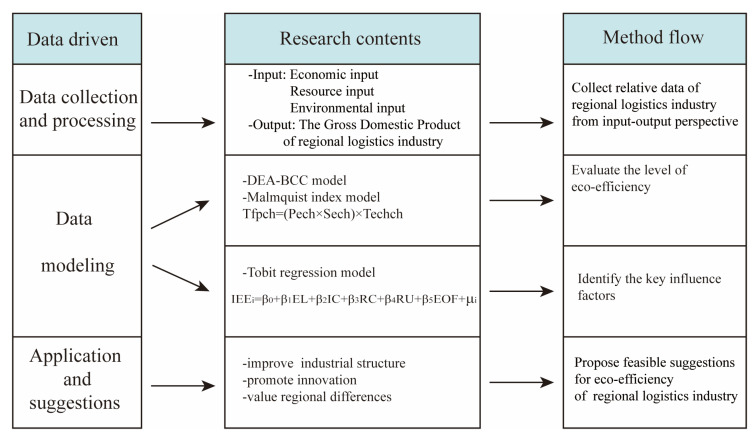
The data-driven eco-efficiency evaluation method flow.

This study builds a data-driven eco-efficiency method to analyze the relationship between resource and environmental inputs and economic outputs of the RLI from a systemic perspective and proposes recommendations to improve the eco-efficiency of the regional RLI from different perspectives. Data collection means collect data on the human and environmental resources invested in the operation of the RLI and the products and services outputted. Data processing is to measure data in different dimensions and units, and data modelling is applied DEA and Malmquist index models for measuring eco-efficiency, and then use Tobit regression models to identify the main influencing factors. Application and suggestion list some targeted measures based on the data-driven approach. Inevitably, there is uncertainty in the above factors, and a data-driven eco-efficiency measurement system can reduce the instability. Therefore, the methodology can provide support for exploration of RLI eco-efficiency for SD of enterprises, supply chains, and industries. Figure 1 is a summary of the method flow.

### 2.2. Data Collection

The data in the ecosystem evaluation index system included input (economy, resources, and environment) and output (expected output) data. These data were mainly collected from the China Logistics Yearbook and Anhui Statistical Yearbook. Data related to economic performance, energy consumption, and environmental pollution in 16 cities in Anhui Province from 2015 to 2020 were collected. Some of the missing data were processed by checking authoritative government and industry reports and via email consultations. When collecting the data, RLI was defined as a complex service industry that combines transport, storage, and postal industries. Moreover, due to the lack of direct data on the environmental inputs of the RLI, the wastewater, gas, and waste emissions of the RLI were indirectly derived by multiplying the ratio of energy consumption of the RLI to industrial energy consumption at different time periods by the industrial wastewater, gas, and waste emissions at the corresponding time periods. Although these indirectly derived data may be less accurate, this collection method is necessary and can be regarded as a supplementary method for data collection.

The multi-dimensionality of eco-efficiency in RLI leads to complexity of the eco-efficiency index system. Any RLI eco-efficiency index system should realize objective comparative analysis in different periods to show the basic situation of the RLI from the aspects of environment, economy, and resources scientifically, reflecting the SD characteristics of low energy consumption, environmental pollution, and high-quality output. Therefore, to be scientific and operable, based on existing research, this study constructed a database that organizes regional logistics eco-efficiency elements, selects input and output indicators from the database, and constructs an RLI eco-efficiency evaluation index system (Table 1). Input indicators include economic, resource, and environmental inputs. Based on previous research [39,40], economic input included indicators from both capital and human input. Resource input was represented by RLI energy consumption. Environmental input included composition of logistics wastewater, waste gas, and solid waste discharge. Additionally, this paper uses the total output value of regional logistics production to represent output index.

### 2.3. Data Processing

#### 2.3.1. Static Measurement of Eco-Efficiency in the RLI

DEA is a data-driven nonparametric efficiency measurement method aimed at maximizing efficiency [7]; it takes the concept of efficiency as the aggregation mode, making efficiency equal to total output divided by total input. The DEA model is used in the efficiency evaluation of DMUs because it does not preset specific function forms and is capable of multiple inputs and outputs. This study applied the DEA–BCC (Banker, Cooper, and Charnes) model (which assumes variable returns to scale and mainly measures the ratio of technical efficiency to scale efficiency) to measure static change in the eco-efficiency of RLI. In our models, K is the number of prefecture-level cities, and each city has m types of input indicators and n types of output indicators. Assuming that there are j decision-making units $DUM_{1},\;DUM_{2}$ ···$DUM_{j}$ to be evaluated, the input indicators are $X_{1}$, $X_{2}$... $X_{j}$ and the output indicators are $Y_{1}$, $Y_{2}$... $Y_{j}$; $x_{ij}\;$ is the i-th input of the logistics unit in j region:

(1) $$\begin{array}{c} X_{j}={\left(x_{1j},x_{2j},\cdots ,x_{mj}\right)}^{T}\\ x_{ij}\geq 0;\;i=1,2\cdots ,m;\;j=1,2,\cdots ,k. \end{array}$$

(2) $$Y_{j}={\left(y_{1j},y_{2j},\cdots y_{nj}\right)}^{T}$$

where $y_{rj}$ is the r-th output of the logistics unit in j region, $y_{rj}\geq 0;\;r=1,2,\cdots ,n.$

The model was applied to evaluate the logistics eco-efficiency of the j-th city:

(3) $$\{\begin{array}{c} min\left[\theta -\varepsilon \left(\overset{m}{\underset{i=1}{\sum }}S^{-}+\overset{m}{\underset{i=1}{\sum }}S^{+}\right)\right]\;\;\;\\ s.t.\overset{k}{\underset{j=1}{\sum }}X_{j}\lambda_{j}+K^{-}=\theta X_{p}\;\;\;\;\;\;\;\;\\ \overset{k}{\underset{j=1}{\sum }}Y_{j}\lambda_{j}-K^{+}=Y_{p}\;\;\;\;\;\;\;\;\;\;\;\;\;\;\\ \overset{k}{\underset{j=1}{\sum }}\lambda_{j}=1\;\;\;\;\;\;\;\;\;\;\;\;\;\;\;\;\;\;\;\;\;\\ S^{-}\geq 0,S^{+}\geq 0,\lambda_{j}\geq 0,j=1,\ldots ,k \end{array}\;\;\;\;\;\;\;$$

where θ is the evaluation value of eco-efficiency, X is the input indicator, Y is the output indicator, $\lambda_{j}$ is the parameter of the decision-making unit, ε is the non-Archimedes infinitesimal parameter, $s^{-}$ is the slack improvement of the input index, and $s^{+}$ is the slack improvement of the output index. When θ = 1, $s^{-}$ ≠ 0, $s^{+}\;$≠ 0, and the DMU is weakly valid for DEA; θ < 1 means the DEA of DMU is invalid, and the situation that θ = 1, *s*^−^ = 0, and $s^{+}$ = 0 represents the DEA of DMU is valid.

The logistics ecological comprehensive efficiency (CE) is equal to the combined effect of pure technical efficiency (PTE) multiplied by scale efficiency (SE):

(4) CE=PTE∗SE  

CE denotes the comprehensive evaluation result of the eco-efficiency of RLI, indicating the minimum input that can be achieved under specified logistics outputs or maximum expected logistics output under the specified inputs. If CE is less than 1, the input and output are relatively ineffective. PTE is the impact of science and technology on logistics eco-efficiency, and SE judges whether there is redundant or insufficient input when developing RLI.

#### 2.3.2. Dynamic Measurement of Eco-Efficiency in the RLI

The DEA–BCC model tends to use panel data to analyze the RLI eco-efficiency of DMU from a static perspective. To combine the static and dynamic analyses, this study introduced the total factor productivity index (Malmquist index). The formula is as follows:

(5) $$M\left(x^{t+1},y^{t+1},x^{t},y^{t}\right)={\left[\frac{B^{t}\left(x^{t+1},y^{t+1}\right)}{B^{t}\left(x^{t},y^{t}\right)}\times \frac{B^{t+1}\left(x^{t+1},y^{t+1}\right)}{B^{t+1}\left(x^{t},y^{t}\right)}\right]}^{\frac{1}{2}}$$

$B^{t}\;$ means the distance function of period t. ($x^{t},y^{t}$) represents the input–output vector of the regional LI in period t. If M > 1, the eco-efficiency of the RLI has risen with the evolution of the period; when M = 1, the eco-efficiency remains unchanged; Additionally, the situation M < 1 signifies that the eco-efficiency has reduced.

The combination of the eco-technical efficiency of RLI (Effch) and the efficiency of ecological technology progress (Techch) is the total factor production efficiency of the RLI. Effch can be calculated using the logistics scale efficiency (Sech) and multiple logistics pure technical efficiency (Pech).

(6) $$Effch=\frac{B^{t}\left(x^{t+1},y^{t+1}\right)}{B^{t}\left(x^{t},y^{t}\right)}$$

(7) $$Techch={\left[\frac{B^{t}\left(x^{t+1},y^{t+1}\right)}{B^{t}\left(x^{t},y^{t}\right)}\times \frac{B^{t+1}\left(x^{t+1},y^{t+1}\right)}{B^{t+1}\left(x^{t},y^{t}\right)}\right]}^{\frac{1}{2}}$$

(8) Tfpch=Effch×techch=(Pech×Sech)×Techch   

#### 2.3.3. Explore Influencing Factors of RLI Eco-Efficiency

The eco-efficiency value of the RLI calculated above is a type of left-truncated data, and applying the ordinary least squares method for linear regression would lead to estimation deviation. The Tobit model [39] is used to solve problems caused by truncated and centralized data. To identify the pivotal influence factors, a two-stage calculation method was selected. First, the DEA model was applied on measuring the efficiency of the DMU; then, the Tobit regression model was used. The specific model is as follows:

(9) $$y^{*}=\beta_{0}+x_{i}\beta +u_{i},\;u_{i}\sim N\left(0,\sigma^{2}\right);\;if\;y_{i}^{*}>0,\;y_{i}^{*}=y_{i};\;if\;y_{i}^{*}\leq 0,\;\;\;\;y_{i}^{*}=0$$

$x_{i}$ represents the explanatory variable, $y^{*}$ is the interpreted variable, $\beta_{0}$ is a constant term, β is a regression parameter vector, and $u_{i}$ (error term) is independent and subject to a normal distribution.

After a comprehensive consideration of the existing research [41,42,43], this study organized five aspects that influence the eco-efficiency of RLI. The mechanism by which each factor affects the eco-efficiency of RLI is as follows:

First, we considered the economic level. As a kind of service industry, the RLI realizes the optimal allocation of social resources by providing services to meet the requirements of varied industries. Cooperation among logistics and other industries jointly promotes the regional economy. However, there is strong relevance between economic progress and environmental pollution, and the developed degree of economy has a complex influence on the eco-efficiency of the RLI. Considering the different population sizes in various prefecture-level cities, this study selected the proportion of each city’s GDP per capita of the province’s GDP per capita to represent the local economic level.

The second factor is the industry structure. The difference in the urban industrial structure reflects the developed degree of the RLI, and the energy structure and industrial energy use intensity of the city affect local eco-efficiency. This study expressed the industrial structure as the ratio of the total value of the RLI to the total value of production of each city.

Third, we considered the research capability. Scientific research capabilities provide technical support for the SD of the RLI. Many studies believe that the intensity of R&D investment and talent reserves are positively related to eco-efficiency. Therefore, this study calculated the ratio of RLI scientific researchers to the total number of scientific researchers in a certain period based on the total number of RLI R&D personnel divided by R&D personnel in that year and then multiplied this ratio by the R&D expenditure of each city to obtain the RLI R&D expenditure of each city, representing the scientific research capacity of each city in different periods.

The fourth factor is resource utilization. To reflect the efficiency of resource utilization in the RLI and measure the transportation situation and economic benefits comprehensively and reasonably, this study selected cargo turnover (actual tonnage of cargo transported × average freight distance), which includes the number of transportation objects and transportation distance, to reflect the transportation production results. With a high level of resource utilization in the RLI, the larger the traffic volume and turnover, the more transportation tasks there are for society. In the context of unreasonable waste of resources (such as repeated transportation and circuitous transportation), the greater the completed traffic volume and turnover volume, the greater the loss and waste to the country.

The fifth and final factor is economic openness to foreign countries. It has been suggested that opening up the regional economy brings more capital and advanced technology and management experience to industry and improves resource utilization efficiency and environmental protection measures; in turn, this should improve eco-efficiency. In this study, we converted the total import and export volume of each city (in USD) into CNY through the exchange rate of the corresponding time period and then used the ratio of the total import and export volume of each city to the GDP of each city to represent the openness of the local economy.

The influencing indicators are presented in Table 2; data are collected from Anhui Provincial Statistical Yearbook, the China Statistical Yearbook, Anhui Provincial Bureau of Statistics, and other institutions for the relevant years.

Based on the index calculation, this study constructed a panel regression model to explore the influence factors of RLI eco-efficiency. The model is as follows:

(10) $$IEE_{i}=\beta_{0}+\beta_{1}EL+\beta_{2}IC+\beta_{3}RC+\beta_{4}RU+\beta_{5}EOF+u_{i}$$

$\;\beta_{0}$, $\beta_{i}$, and $u_{i}$ represent the regression constant, the coefficient, and random error.

### 2.4. Data Application

Data-driven refers to data-centered decision-making and action. Specifically, it refers to formation of an automatic decision-making model through training and fitting based on collecting, integrating, refining a large amount of data, and organizing information. This paper collects the input and output data of RLI economy, resources, and environment comprehensively, brings in the DEA and Malmquist index models for measuring eco-efficiency and the Tobit regression model for identifying the main influencing factors of RLI eco-efficiency, and evaluates, identifies, and improves the eco-efficiency of RLI after internalizing the data.

The first step is to input data, select 7 inputs and 1 expected output, including economy, resources, and environment, and build the RLI eco-efficiency factor database and the eco-efficiency evaluation index system.

The second step is to use DEA–BCC model and Malmquist index model, measuring the eco-efficiency of RLI and the change in eco-efficiency of RLI from a static perspective and from a dynamic perspective, respectively. Then, use the Tobit model to reveal the correlation and influence effect between influence factors and RLI eco-efficiency.

Third step, put forward targeted policy recommendations to improve the eco-efficiency of the RLI according to the measurement results and the identified main influencing factors.

## 3. Case Study

### 3.1. Background

Anhui Province, which has 16 prefecture-level cities, is located in the Yangtze River Delta; it is surrounded by Jiangsu, Zhejiang, Hunan, and Hubei provinces. Anhui Province is a strategic hub for national economic development and represents the central area for several major domestic economic sectors. Jiangsu, Zhejiang, Shanghai, and Anhui have strong consumption capacities, large population densities, strong commercial demand, intensive logistics networks, and mature logistics ecological chains. According to data released by the Anhui Federation of Logistics and Procurement, the total amount of social logistics in Anhui was CNY 8082.26 billion in 2021, up 15.1% year on year. Simultaneously, logistics costs declined dramatically. The total cost of social logistics in the province was CNY 602.7 billion, an increase of 7.3%. The ratio of total social logistics costs to GDP is 14.0%, which is 0.6% lower than that of the national level.

However, accompanied with the development of economy, the problem of inefficient utilization of resources and environmental pollution is more and more serious. This study evaluates and explores the influencing factors of the eco-efficiency of the RLI and then puts forward targeted policy recommendations.

### 3.2. Static Eco-Efficiency of RLI in 16 Prefecture-Level Cities

Using Formulas (1)–(4) [DEA–BCC model], the scale efficiency (SE), pure technical efficiency (PTE), and comprehensive efficiency (CE) from 2015 to 2020 in Anhui Province were calculated. As CE = PTE × SE, comprehensive efficiency is the most representative; thus, in this study, we considered comprehensive efficiency as the key analysis object and the results are listed in Table 3.

**Figure 2:**
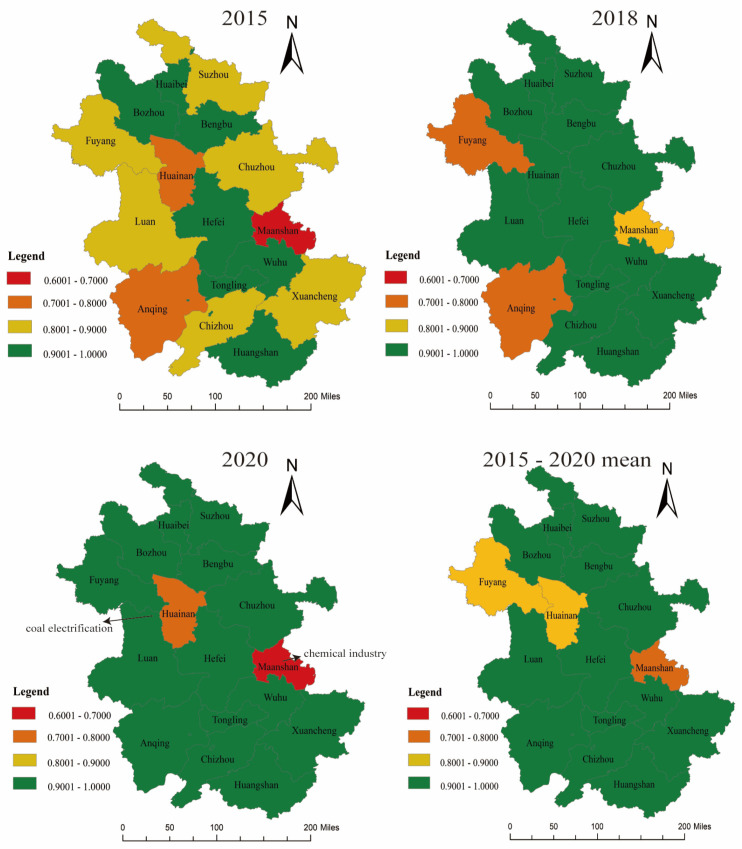
Comparison of mean comprehensive efficiency of Anhui Province in different years.

As shown in Table 3, the comprehensive efficiency of Anhui Province grew rapidly from 2015 to 2016, rose over the next 3 years, peaked in 2019, and then decreased to 0.955 by 2020. As such, overall, the eco-efficiency of Anhui Province has improved over the past 5 years. However, differences among provincial-level cities are evident (Figure 2). From 2015 to 2020, Bengbu, Huainan, and Maanshan performed non-DEA effectively, and the input–output ratio was less efficient. Hefei, Bozhou, Wuhu, Tongling, and Huangshan had more rational input–output ratios.

The reasons for the calculation result (Table 3) are that these cities have obvious weaknesses in their industrial and energy structures. The proportion of traditional industries is large, whereas the proportion of logistics and other tertiary industries is small.

Specifically, the chemical industry in Maanshan is well-developed. The raw materials and industrial waste of toxic and harmful substances produced in production processes increase the risk of soil and groundwater pollution, which accumulates over long time periods. Moreover, some enterprises only pursue economic benefits, do not use pollution control facilities normally, and illegally dispose of hazardous waste, which greatly hinders the eco-efficiency of Maanshan.

Bengbu is the largest traditional industrial base in Anhui Province (emerging industries occupy a small proportion), and there is a large stock of highly polluting and energy-consuming industries, such as the chemical industry, printing and dyeing, glass, and brewing. Moreover, it is difficult to make dramatic changes to the energy structure in the short term when fossil fuels occupy an absolute dominant position.

The industrial and energy structures of Huainan are characterized by high carbon content. Huainan’s industrial structure is biased towards coal electrification. Thermal power accounts for >75% of the city’s total coal consumption; however, the coal electrification industry emits a large amount of CO_2_, and the energy consumption per unit GDP of Huainan City (0.637 tons of standard coal/CNY 10,000) is far greater than the average level of neighboring cities and Anhui Province (0.468 tons of standard coal/CNY 10,000). With tightening of resource and environmental constraints, the contradictions between economy and insufficient ecological environment carrying capacity will continue to exist.

### 3.3. Dynamic Eco-Efficiency of RLI

**Figure 3:**
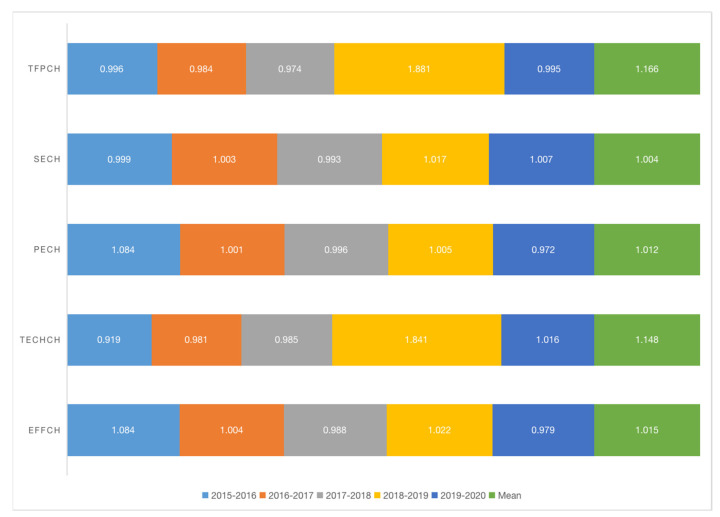
Malmquist index and decomposition of RLI eco-efficiency in Anhui from 2015 to 2020.

The Malmquist index model was selected to measure dynamic trends of RLI eco-efficiency in Anhui Province. The RLI comprehensive efficiency (Effch), technological progress efficiency (Techch), scale efficiency (Sech), pure technical efficiency (Pech), and total factor eco-efficiency (Tfpch) of Anhui Province were calculated using Formulas (5)–(8), and the results are demonstrated in Figure 3.

As shown in Figure 3, the mean Tfpch was 1.166, illustrating that the overall Tfpch is increasing despite slight downward trends in 2015–2018 and after reaching the peak (1.881) in 2018–2019. This trend is because, compared with other Yangtze River Delta regions, Anhui Province has a relatively weak economic foundation, the RLI started late, the development model was highly dependent on energy consumption, and there was a large proportion of road transport, all of which contributed to the downward trends.

Anhui Province has abundant inland water transport resources, and its waterway mileage ranks eighth in China. However, owing to a long-term “Small scale, scattered layout and weak economic strength” pattern, homogeneous competition and disordered shoreline development are prominent; the natural resource advantage of the waterway has not been transformed into a development advantage. In 2019, Anhui Province integrated port and shipping resources, connecting the Hefei and Wuhu ports effectively, to minimize costs and improve efficiency. Based on the 5-year data, Sech and Pech are weaker than the other indicators. According to Formula (8), lower Sech and Pech affect Effch and Tfpch. Therefore, Anhui Province should improve the management level and technology of logistics enterprises and expand their scale.

**Figure 4:**
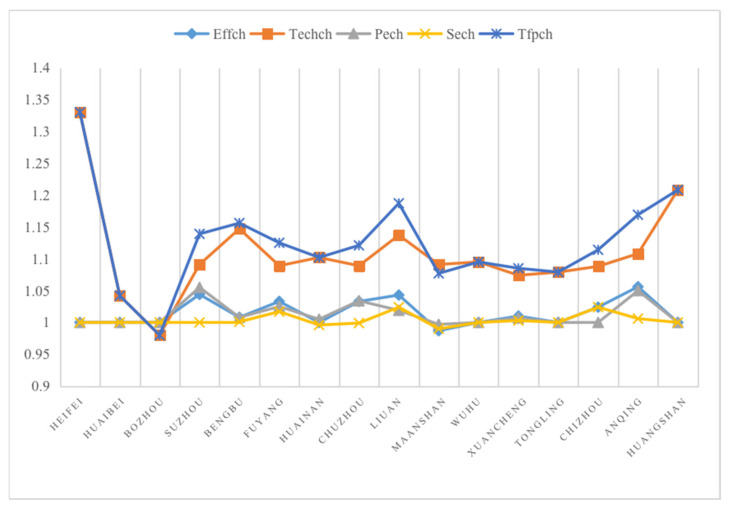
Malmquist indices of 16 cities in Anhui Province from 2015 to 2020.

The Malmquist index and decomposition of eco-efficiency for the RLI of 16 cities are shown in Table 4 and Figure 4. From the perspective of Tfpch, except for Bozhou, the RLI eco-efficiency improved from 2015 to 2020. However, Bozhou’s relative efficiency decreased, and Tfpch showed a downward trend, mainly because Bozhou’s technological development is low. Therefore, local governments should focus on promoting environmental protection technology in the LI. Moreover, the Sech values of Huanan, Suzhou, and Maanshan were <1, particularly Maanshan, whose Effch (which is influenced by both Pech and Sech), Pech, and Sech were <1. Therefore, local governments should reorganize and build large-scale logistics and distribution centers to promote development of a joint scale to improve Sech. Enterprises mainly improve Pech by improving production technology level and service quality.

### 3.4. Influencing Factors for RLI Eco-Efficiency in Anhui Province

To identify the reasons for the horizontal and vertical discrepancy in RLI efficiency among the different cities, this study selected the Tobit model (Table 2) to identify the influencing factors. This was applied using Stata software, which conducted random effect regression analysis and calculated the impact size and path of the influencing factors of eco-efficiency. The calculation results are presented in Table 5.

The *p*-value of economic level was 0.084 < 0.1, with a significance level of 10%, proving that the degree of regional economic development has a slight reverse effect on the improvement in eco-efficiency in the RLI. Industry construction passed the 5% significance level test, correlating with eco-efficiency negatively and indicating that the inhibitory effect of industrial structure on the eco-efficiency of the RLI is not significant. Research capability was significant (significance level of 10%), correlating with RLI eco-efficiency positively and meaning that increasing investment in scientific education and R&D of relevant local disciplines can promote high-quality development of the RLI. The significance of resource utilization and logistics eco-efficiency was <5%, showing a significant correlation, but the coefficient was −3.93 × 10^−8^, indicating that the improvement in resource utilization efficiency only has a very limited effect on improving RLI eco-efficiency. The *p*-value of economic openness to foreign capital was 0.421 > 0.1, failing to pass the significance level test, representing that the measures to improve the regional RLI eco-efficiency by introducing foreign capital are not significant.

### 3.5. Policy Suggestions

In summary, from 2015 to 2020, the overall ecological comprehensive efficiency of the RLI demonstrated a fluctuating upward tendency, but the average value did not reach the DEA efficiency, and variation among different cities was obvious. Bengbu, Huanan, and Maanshan faced challenges in improving eco-efficiency owing to their industrial and energy structures. The eco-efficiency of the RLI has improved slightly over the past 5 years, and the overall trend was significantly affected by efforts and technology. Affected by various factors, Tfpch in some cities was lower than the provincial average level. Therefore, to promote the overall factor eco-efficiency of the RLI in each city, implementing policies based on local conditions and providing precise policy support based on quantitative measurement results are essential. Through empirical analysis, the economic level, industry construct, research capability, and resource utilization were correlated with RLI eco-efficiency in Anhui Province. Economic openness to foreign factors does not significantly influence the RLI eco-efficiency.

To improve the RLI eco-efficiency and promote sustainability, this study offers several suggestions.

First, there is a need to exploit the internal demand of RLI to promote transformation of industrial structure. Owing to the impact of COVID-19, the total demand of industry is relatively low. Therefore, it is necessary to tap the potential of domestic demand, expand the business scope of the RLI, and stimulate consumer demand, such as by developing high-end business lines in the logistics service industry. It is also crucial to increase support for key areas, such as ecological and environmental protection, and to improve the quality of service industry development steadily.

Second, there is a need to promote innovation and cluster driven digital empowerment. Innovation is a power source for the long-term SD of the RLI. Therefore, local government should improve the education level of the overall population of Anhui Province, increase introduction and training of high-end talent, and create an environment suitable for innovation-driven SD of the RLI. Moreover, the proportion of R&D investment in the GDP should be increased, and industrial innovation centers should be built, attracting universities, research institutes, R&D institutions, and logistics enterprises to improve the innovation incentive mechanism. This measurement can open up industrialization channels for scientific and technological achievements, increase the utilization efficiency of various innovative resources, and accelerate the transformation of achievements.

Finally, there is a need to attach importance to regional differences and promote new energy for the industrial sustainability. Use of coal and other energy sources is widespread owing to the dominance of traditional industries in some cities. Therefore, application of new energy should consider the logistics of local industrial conditions. Transport vehicles using petroleum energy should be phased out and new energy transport vehicles should be introduced. Enterprises should be encouraged to use electronic express bills; increase use of green recyclable materials in the process of express delivery, transportation, distribution, and sorting; recycle express packaging; reduce repeated packaging and excessive packaging; and reduce the cost of enterprises in packaging and transportation.

## 4. Conclusions

With fierce competition in the global market, rapid development of the RLI has consumed a significant amount of energy and caused pollution, hindering the SD of the economy. To improve eco-efficiency, this paper proposed a data-driven method to evaluate and enhance regional eco-efficiency. In this study, the DEA–BCC model and Malmquist index model were applied to evaluate eco-efficiency from static and dynamic perspectives, respectively, and then assess the current eco-efficiency levels of the RLI in specific regions and the change tendency of total-factor eco-efficiency. Finally, the Tobit regression model was applied to explore the key influence factors.

The main innovations are as follows. When selecting research objects and data, regions with development potential and research value were prioritized, considering differences in existing industrial development models. Ultimately, this paper conducted a comparative study on the eco-efficiency logistics in Anhui Province. Second, regional logistics eco-efficiency was assessed based on multidimensional data using a data-driven approach, which ensured the objectivity of the measurement and identification results. Third, this study analyzed the correlation and significance of various influencing factors of RLI eco-efficiency in different areas and put forward policy suggestions on RLI eco-efficiency in each city on this basis.

From the perspective of practitioners, data-driven approaches better serve the government’s precise implementation and RLI’s precise management. To improve eco-efficiency and control environmental costs, it is crucial to strengthen research, development, and application of ecological technologies in the RLI; promote use of new energy transport vehicles with electronic express bills; make full use of the data-driven methods to compute transport routes with high resource utilization efficiency; strengthen total management of production input factors in the RLI; and comprehensively improve resource utilization efficiency and resource recycling in the RLI.

In terms of governance, governments in different regions should formulate targeted economic development goals and emissions targets according to the situation of local enterprises and relevant research and publicly ensure that enterprises and people understand the links between economic development and ecology. Under the guidance of carbon neutrality goals, the RLI should seek to systematically reduce energy and resource inputs and improve its eco-efficiency.

The shortcomings of this study are that the interaction between different indicators is not fully considered when modelling and calculating the influencing factors. Therefore, in future research, the interaction between influencing factors will be considered.

## Figures and Tables

**Table 1:** Eco-efficiency evaluation index system for RLI.

Level I Indicator	Level II Indicator	Level III Indicator	Unit
Input	Economic input	X_1_ Fixed assets investment in LI	CNY 100 million
		X_2_ Number of trucks	10,000 cars
		X_3_ Number of employees in LI	10,000 people
	Resource input	X_4_ Energy consumption of LI	10,000 tons of standard coal
	Environmental input	X_5_ Total wastewater discharge of LI	10,000 tons
		X_6_ Sulfur dioxide emission of LI	10,000 tons
		X_7_ Solid waste generated by LI	10,000 tons
Output	Expected output	Y_1_ GDP of LI	CNY 100 million

**Table 2:** Main influencing indicators of RLI eco-efficiency.

Statistical Variable	Variable	Code	Calculation Method
Y	Eco-efficiency of RLI	LEE	Comprehensive eco-efficiency of RLI
A_1_	Economic level	EL	Each city’s GDP per capita/province’s GDP per capita
A_2_	Industry structure	IC	Total value of RLI/total value of production
A_3_	Research capability	RC	RLI R&D expenditure
A_4_	Resource utilization	RU	Actual tonnage of cargo transported × average freight distance
A_5_	Economic openness to foreign	EOF	Total import and export volume of each city/GDP of each city

**Table 3:** Comprehensive eco-efficiency of the RLI in Anhui Province (2015–2020).

	City	2015	2016	2017	2018	2019	2020	Mean
1	Heifei	1	1	1	1	1	1	1.00000
2	Huaibei	1	0.934	1	0.948	1	1	0.98033
3	Bozhou	1	1	1	1	1	1	1.00000
4	Suzhou	0.805	1	0.897	1	1	1	0.95033
5	Bengbu	0.96	0.989	1	1	1	1	0.99150
6	Fuyang	0.851	0.799	0.737	0.745	1	1	0.85533
7	Huainan	0.753	0.823	1	1	0.974	0.754	0.88400
8	Chuzhou	0.823	1	1	1	1	0.969	0.96533
9	Liuan	0.808	1	1	1	1	1	0.96800
10	Maanshan	0.655	0.852	0.925	0.809	0.628	0.614	0.77100
11	Wuhu	1	1	1	1	1	1	1.00000
12	Xuancheng	0.895	0.981	0.95	0.921	0.965	0.943	0.94250
13	Tongling	1	1	1	1	1	1	1.00000
14	Chizhou	0.889	0.933	1	1	1	1	0.97033
15	Anqing	0.762	1	0.868	0.796	1	1	0.90433
16	Huangshan	1	1	1	1	1	1	1.00000
	Mean	0.88756	0.95694	0.96106	0.95119	0.99593	0.95500	0.95128

**Table 4:** Malmquist index and decomposition of eco-efficiency of 16 cities in Anhui Province from 2015 to 2020.

Malmquist Index Summary of Firm Means					
Firm	Effch	Techch	Pech	Sech	Tfpch
Heifei	1	1.33	1	1	1.33
Huaibei	1	1.042	1	1	1.042
Bozhou	1	0.98	1	1	0.98
Suzhou	1.044	1.091	1.055	1	1.139
Bengbu	1.008	1.147	1.008	1.001	1.156
Fuyang	1.033	1.089	1.025	1.017	1.125
Huainan	1	1.102	1.005	0.996	1.102
Chuzhou	1.033	1.089	1.034	0.999	1.121
Liuan	1.043	1.137	1.019	1.024	1.187
Maanshan	0.987	1.091	0.997	0.99	1.077
Wuhu	1	1.095	1	1	1.095
Xuancheng	1.01	1.074	1.007	1.003	1.085
Tongling	1	1.079	1	1	1.079
Chizhou	1.024	1.088	1	1.024	1.114
Anqing	1.056	1.108	1.05	1.006	1.169
Huangshan	1	1.208	1	1	1.208

**Table 5:** Tobit regression results.

var9	Coef.	Std.E	z	*p* > z	[95%Conf.Interval]	
el	−0.3617489	0.2094709	−1.73	0.084 *	−0.7723044	0.0488066
ic	−0.0000313	0.0000123	−2.54	0.011 **	−0.0000555	−7.12 × 10^−6^
rc	0.0001767	0.0001001	1.77	0.078 *	−0.0000195	0.0003728
ru	−3.93 × 10^−8^	1.84 × 10^−8^	−2.13	0.033 **	−7.54 × 10^−8^	−3.14 × 10^−9^
eof	0.6339006	0.7872579	0.81	0.421	−0.9090966	2.176898
_cons	1.391836	0.1847201	7.53	0	1.029791	1.753881

** and * indicate passing the test at 5%, and 10% statistical levels, respectively.

## Data Availability

All data generated or analyzed during this study are included in this published article.
